# Supplementary material for: Immune correlates of early clearance of Mycobacterium tuberculosis among tuberculosis household contacts in Indonesia
Source: Nat Commun. 2025 Jan 2;16:309. doi: 10.1038/s41467-024-55501-6 (PMC11695729; doi:10.1038/s41467-024-55501-6)
Supplement: Supplementary file 1 — Supplementary Information [file 41467_2024_55501_MOESM1_ESM.pdf]

**Table S1**. Clinical characteristics of IGRA-negative tuberculosis household contacts

| | Total | | | Strict cut-off[d] | | |
|---|---|---|---|---|---|---|
| | **IGRA converters**[a] (N = 116) | **Persistently IGRA-negatives**[a] (N = 317) | ***P* value**[b] | **True IGRA converters**[a] (N = 51) | **True persistently IGRA-negatives**[a] (N = 237) | ***P* value**[b] |
| **Case contact characteristics** | | | | | | |
| Age | 23 (15 – 36) | 22 (12 – 40) | 0.8 | 19 (11 – 32) | 21 (11 – 38) | 0.5 |
| Female sex | 51% | 54% | | 57% | 54% | |
| Presence of BCG scar | 74% | 87% | <0.001 | 69% | 90% | <0.001 |
| Current and previous smoking | 36% | 29% | 0.2 | 31%) | 25% | 0.4 |
| BMI, kg/$m^2$ | 21.0 (17.7 – 24.8) | 20.0 (16.7 – 24.3) | 0.12 | 19.8 (16.6 – 23.9) | 19.9 (16.5 – 24.2) | >0.9 |
| Diabetes[c] | 5.2% | 3.5% | 0.5 | 3.9% | 3.8% | >0.9 |
| **Exposure to the index case** | | | | | | |
| Exposure risk score[e] | 0.32 (0.23 – 0.38) | 0.25 (0.17 – 0.32) | <0.001 | 0.32 (0.25 – 0.38) | 0.23 (0.17 – 0.30) | <0.001 |
| Sleeping in the same room as the index case | 19% | 20% | 0.8 | 18% | 16% | 0.7 |
| Waking hours spent with the index case a day before enrollment | 5 (3 – 8) | 3 (1 – 7) | 0.003 | 4 (3 – 9) | 3 (1 – 6) | 0.026 |
| Index case highest smear grade | | | <0.001 | | | <0.001 |
| *Scanty* | 2.6% | 10% | | 0% | 10% | |
| *1+* | 21% | 31% | | 14% | 35% | |
| *2+* | 24% | 26% | | 27% | 25% | |
| *3+* | 53% | 33% | | 59% | 30% | |
| Presence of cavities on chest x-ray of index | 50% | 42% | 0.2 | 48% | 38% | 0.2 |
| Extent of x-ray abnormalities | 40 (25 – 55) | 35 (20 – 60) | 0.6 | 40 (25 – 64) | 35 (25 – 55) | 0.7 |
| *M. tuberculosis* L2 (Beijing) strain in the index case | 36% | 25% | 0.028 | 40% | 23% | 0.016 |
| Blood count parameters at baseline | | | | | | |
| Hemoglobin g/dL | 14.15 (13.10 – 15.43) | 13.60 (12.80 – 14.90) | 0.024 | 13.90 (12.95, 14.85) | 13.50 (12.80, 14.60) | 0.4 |
| Platelets 1,000/$mm^3$ | 298 (249 – 362) | 306 (261 – 359) | 0.8 | 305 (267 – 370) | 308 (266 – 362) | 0.7 |
| Leukocytes 1,000/$mm^3$ | 7.55 (6.20 – 8.63) | 7.40 (6.30 – 8.50) | >0.9 | 7.60 (6.40 – 8.50) | 7.30 (6.20 – 8.50) | 0.7 |
| Lymphocytes 1,000/µL | 2.56 (2.13 – 3.13) | 2.64 (2.08 – 3.06) | >0.9 | 2.72 (2.20 – 3.17) | 2.61 (2.02 – 3.02) | 0.2 |
| Neutrophiles 1,000/µL | 4.12 (3.20 – 5.04) | 3.91 (3.20 – 5.01) | 0.8 | 4.16 (3.28 – 4.85) | 3.90 (3.24 – 4.99) | >0.9 |
| Monocytes 1,000/µL | 0.42 (0.33 – 0.54) | 0.43 (0.31 – 0.57) | 0.6 | 0.42 (0.34 – 0.53) | 0.43 (0.32 – 0.57) | 0.6 |
| Quantitative IFNγ release assay result | | | | | | |
| IFNγ Nil tube IU/L | 0.15 (0.08 – 0.31) | 0.13 (0.08 – 0.25) | 0.3 | 0.14 (0.07, 0.32) | 0.13 (0.08 – 0.24) | 0.5 |
| IFNγ TB-Nil tube IU/L | 0.08 (-0.01 – 0.23) | 0.01 (-0.02 – 0.08) | <0.001 | 0.00 (-0.08, 0.04) | 0.00 (-0.04 – 0.03) | 0.7 |
| IFNγ Mitogen-Nil tube IU/L | 8.08 (3.34 – 10.00) | 8.91 (3.42 – 10.00) | 0.5 | 8.93 (4.20, 10.00) | 8.51 (3.05 – 10.00) | 0.7 |

Abbreviations: BCG, Bacillus Calmette-Guerin; BMI, body mass index; IQR, interquartile range.

[a] Median (IQR); %
[b] Mann-Whitney U test; Pearson's Chi-squared test; Fisher's exact test
[c] Diabetes defined as follows: no diabetes, random capillary blood glucose >101 mg/dL or hemoglobin A1c (HbA1c) <5.7%; prediabetes, HbA1c 5.7%–6.4%; diabetes, HbA1c ≥6.5.
[d] Subset using strict IGRA cutoff of <0.15 IU/mL as negative and >0.70 IU/mL as positive result instead of the cutoff provided in the kit (0.35 IU/mL)
[e] Exposure risk scores were derived from a logistic regression model of *Mtb* exposure variables (index case: sputum smear grade, cavities, extent of x-ray disease; contacts: hours spent with and sleeping proximity to the case) with IGRA results at 14 weeks as the dependent variable[1]

## Table S2. Relative Risk of IGRA conversion in contacts exposed to different strain and the effect of BCG vaccination using different cut-off criteria

**A**

| **Relative Risk of IGRA conversion in contacts with manufacturer cut-off by index case *Mtb* genotype** | | | | | | |
|---|---|---|---|---|---|---|
| Genotype | Persistently IGRA-negatives n=275 | IGRA converters n=108 | Relative Risk (95% CI) | *P* value | Adjusted RR (95% CI) | *P* value |
| Other | 206 (75%) | 69 (63%) | 1.00 (ref) | | 1.00 (ref) | |
| L2 (Beijing) | 69 (25%) | 39 (37%) | 1.44 (0.98-2.10) | <0.001 | 1.39 (1.00-1.93) | 0.048 |

| **Relative Risk of IGRA conversion in contacts *with strict cut-off* by index case *Mtb* genotype** | | | | |
|---|---|---|---|---|
| Genotype | Persistently IGRA-negatives n=206 | IGRA converters n=50 | Relative Risk (95% CI) | *P* value |
| Other | 158 (76%) | 30 (60%) | 1.00 (ref) | |
| L2 (Beijing) | 48 (24%) | 20 (40%) | 1.84 (1.11-2.97) | 0.015 |

**B**

| **Relative Risk of IGRA conversion by contacts BCG vaccination status and index case *Mtb* genotype** | | | | | | | |
|---|---|---|---|---|---|---|---|
| Genotype | BCG | Persistently IGRA-negatives n=275 | IGRA converters n=108 | Relative Risk (95% CI) | *P* value | Adjusted RR (95% CI) | *P* value |
| Other | No | 22 (10%) | 21 (30%) | 1.00 (ref) | | 1.00 (ref) | |
| | Yes | 184 (90%) | 48 (70%) | 0.42 (0.28-0.63) | <0.001 | 0.40 (0.27-0.61) | <0.001 |
| L2 (Beijing) | No | 13 (19%) | 7 (18%) | 1.00 (ref) | | 1.00 (ref) | |
| | Yes | 56 (81%) | 32 (82%) | 1.04 (0.54-2.01) | 0.9 | 1.02 (0.56-1.85) | 0.9 |

| **Relative Risk of IGRA conversion with strict IGRA cut-off by contacts BCG vaccination status and index case *Mtb* genotype** | | | | | |
|---|---|---|---|---|---|
| Genotype | BCG | Persistently IGRA-negatives n=206 | IGRA converters n=50 | Relative Risk (95% CI) | *P* value |
| Other | No | 14 (10%) | 12 (30%) | 1.00 (ref) | |
| | Yes | 144 (90%) | 18 (70%) | 0.24 (0.13-0.43) | <0.001 |
| L2 (Beijing) | No | 6 (13%) | 4 (20%) | 1.00 (ref) | |
| | Yes | 42 (87%) | 16 (80%) | 0.69 (0.29-1.63) | 0.4 |

**Table S3**. Characteristics of household contacts with anti-*Mtb* antibodies measured

| | **Baseline IGRA-positive**[a] (N = 100) | **Baseline IGRA-negative**[a] (N = 433) | ***P* value**[b] |
|---|---|---|---|
| **Case contact characteristics** | | | |
| Age | 30 (14 – 46) | 22 (12 – 39) | 0.069 |
| Female sex | 60% | 53% | 0.20 |
| Presence of BCG scar | 79% | 84% | 0.25 |
| Smoking | 32% | 31% | 0.21 |
| BMI, kg/$m^2$ | 20.8 (16.8 – 24.2) | 20.2 (16.8 – 24.4) | 0.72 |
| Diabetes[c] | 2.0% | 3.9% | 0.57 |
| Exposure to the index case | | | |
| Sleeping in the same room as the index case | 34% | 20% | 0.002 |
| Waking hours spent with the index case a day before enrollment | 6.0 (2.0 – 10.0) | 4.0 (1.0 – 8.0) | 0.006 |
| Index case highest smear grade | | | 0.17 |
| *Scanty* | 3.0% | 8.3% | |
| *1+* | 24% | 28% | |
| *2+* | 27% | 25% | |
| *3+* | 46% | 38% | |
| Presence of cavities on chest x-ray of index | 58% | 44% | 0.011 |
| Extent of x-ray abnormalities | 45 (25 – 66) | 40 (25 – 59) | 0.24 |
| *M. tuberculosis* L2 (Beijing) strain in the index case | 26% | 28% | 0.76 |
| Blood count parameters at baseline | | | |
| Hemoglobin g/dL | 13.55 (12.65 – 14.70) | 13.70 (12.80 – 15.00) | 0.17 |
| Platelets 1,000/$mm^3$ | 289 (249 – 339) | 305 (258 – 360) | 0.16 |
| Leukocytes 1,000/$mm^3$ | 7.45 (6.48 – 8.50) | 7.40 (6.20 – 8.60) | 0.91 |
| Lymphocytes 1,000/µL | 2.70 (2.15 – 3.26) | 2.60 (2.12 – 3.07) | 0.17 |
| Neutrophiles 1,000/µL | 3.98 (3.29 – 4.81) | 4.03 (3.20 – 5.02) | 0.81 |
| Monocytes 1,000/µL | 0.41 (0.30 – 0.55) | 0.43 (0.32 – 0.56) | 0.75 |
| Quantitative IFNγ release assay result at baseline | | | |
| IFNγ Nil tube IU/L | 0.15 (0.10 – 0.30) | 0.14 (0.08 – 0.28) | 0.24 |
| IFNγ TB-Nil tube IU/L | 2.22 (0.95 – 6.75) | 0.02 (-0.02 – 0.13) | <0.001 |
| IFNγ Mitogen-Nil tube IU/L | 10.00 (3.72 – 10.00) | 8.68 (3.41 – 10.00) | 0.31 |

Abbreviations: BCG, Bacillus Calmette-Guerin; BMI, body mass index; IQR, interquartile range.

[a] Median (IQR); %
[b] Mann-Whitney U test; Pearson's Chi-squared test; Fisher's exact test
[c] Diabetes defined as follows: no diabetes, random capillary blood glucose >101 mg/dL or hemoglobin A1c (HbA1c) <5.7%; prediabetes, HbA1c 5.7%–6.4%; diabetes, HbA1c ≥6.5.

**Table S4**. Characteristics of BCG-vaccinated volunteers with antibody and PBMC stimulation measurements

| Characteristic | N = 298[1] |
|---|---|
| Age[1] | 23 (18 – 71) |
| Female | 56% |
| Body mass index[2] | 22.15 (20.80 – 23.62) |

[1] Median (range)
[2] Median (IQR)

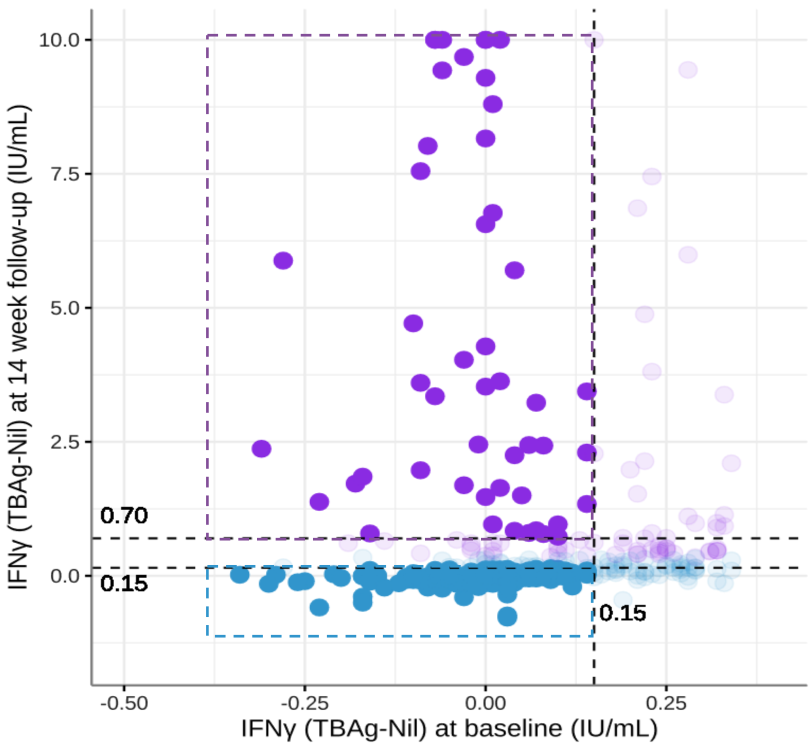


**Supplementary Figure 1. Subset of persistently IGRA-negative individuals and IGRA converters using stricter IGRA cutoff**

For IGRA negative individuals at baseline (N=433), we used a strict cut-off value for TB – Nil IFNγ of less than 0.15 IU/mL (both at baseline and 14 weeks), to classify subjects as persistently IGRA-negative (N=237, blue dotted box), and < 0.15 IU/mL at baseline and > 0.7 at 14 weeks to classify subjects as IGRA-converters (N=51, purple).

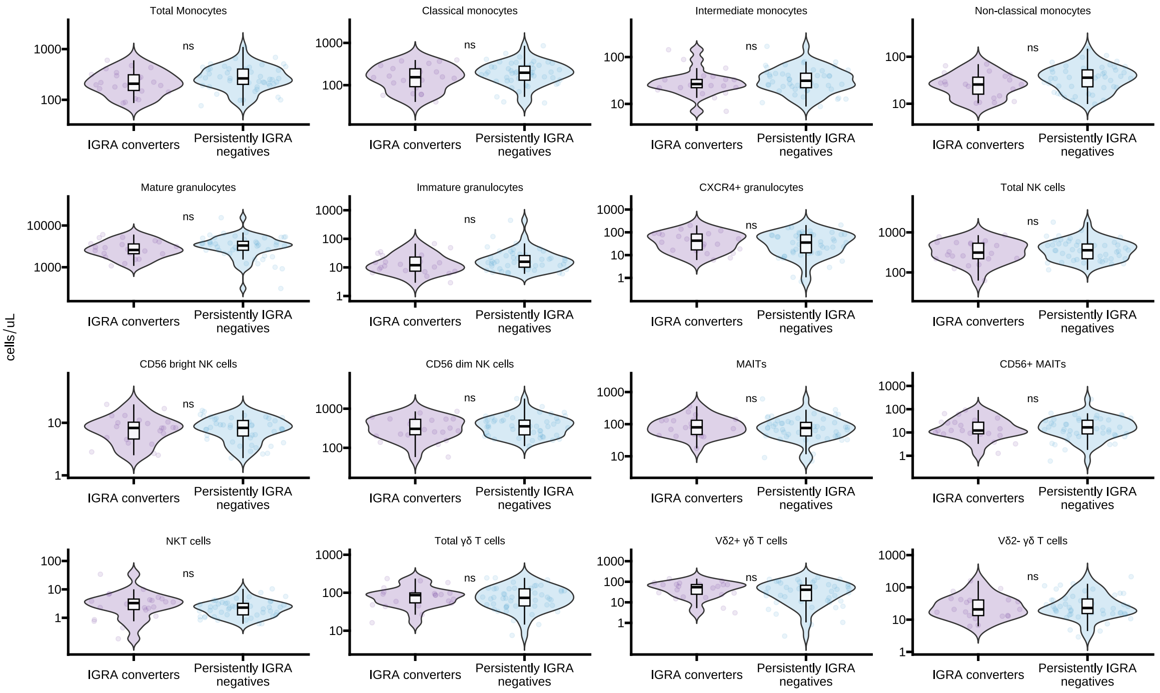


**Supplementary Figure 2. Innate immune cells in IGRA converters and persistently IGRA-negative individuals**

In IGRA negative individuals with complete flow-cytometric measurements, persistently IGRA-negative individuals (N = 48) and IGRA converters (N = 22), frequencies of circulating innate immune cells (numbers / μL blood) were compared at week 2. There was no difference between IGRA converters and persistently IGRA-negative individuals in individual innate immune cells (Mann-Whitney U test after Benjamini-Hochberg correction for multiple testing).

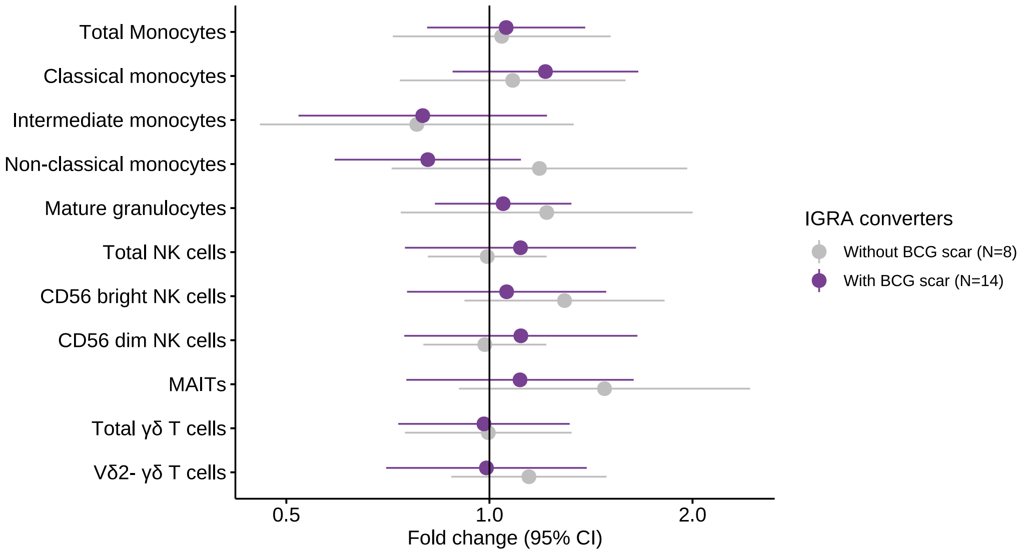


**Supplementary Figure 3**. **Innate immune cell population analysis of IGRA converters with and without BCG scar.**

In IGRA negative individuals with a BCG scar and complete flow-cytometric measurements, IGRA converters with BCG scar (N = 14) and without BCG scar (N = 8), frequencies of circulating innate immune cells (numbers / μL blood) were compared between week 2 and week 14 and shown here as fold change from week 14 to week 2. In IGRA converters both with and without BCG scar, no significant differences were seen (paired wilcoxon signed rank tests after Benjamini-Hochberg correction for multiple testing).

**A**

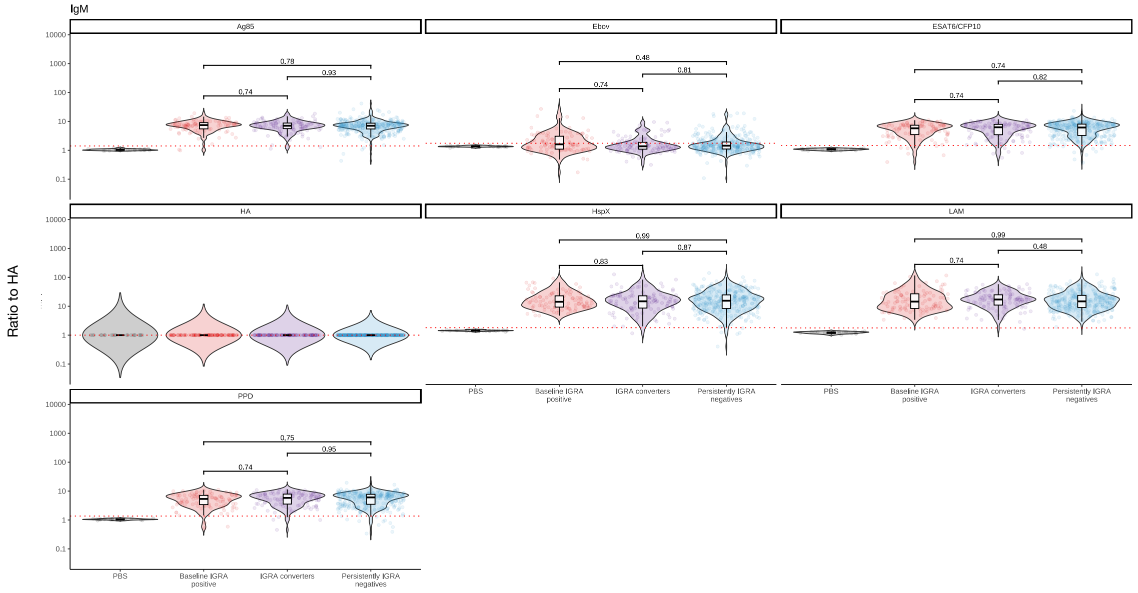


**Supplementary Figure 4. Antibody level as ratio to HA in all groups and PBS control**

Antibody levels ($log_{10}$ transformed), standardized to the positive control influenza virus hemaglutinnin (HA) as a ratio of MFI / MFI of anti-HA antibodies. To improve specificity (signal-noise ratio) the mean standardized antibody level + 6SD (standard deviation) in the PBS control was used as a cutoff (dotted lines), for IgM (**A**), total IgG (**B**), IgG1 (**C**), IgG2 (**D**), IgG3 (**E**), IgA1 (**F**), IgA2 (**G**), FcγR (**H-K**). Based on this cut-offs, we only included measurements of 5 antibody isotypes (IgM, IgG2, IgG3, IgA1, IgA2) against 5 different *Mtb* antigens in subsequent analysis. The antibody level as ratio to HA for Ebola-specific antibodies, as a negative control, were below the mean ratio to HA + 6 SD in PBS. All comparison were done using Mann-Whitney U test with correction for multiple testing using Benjamini-Hochberg (FDR).

**B**

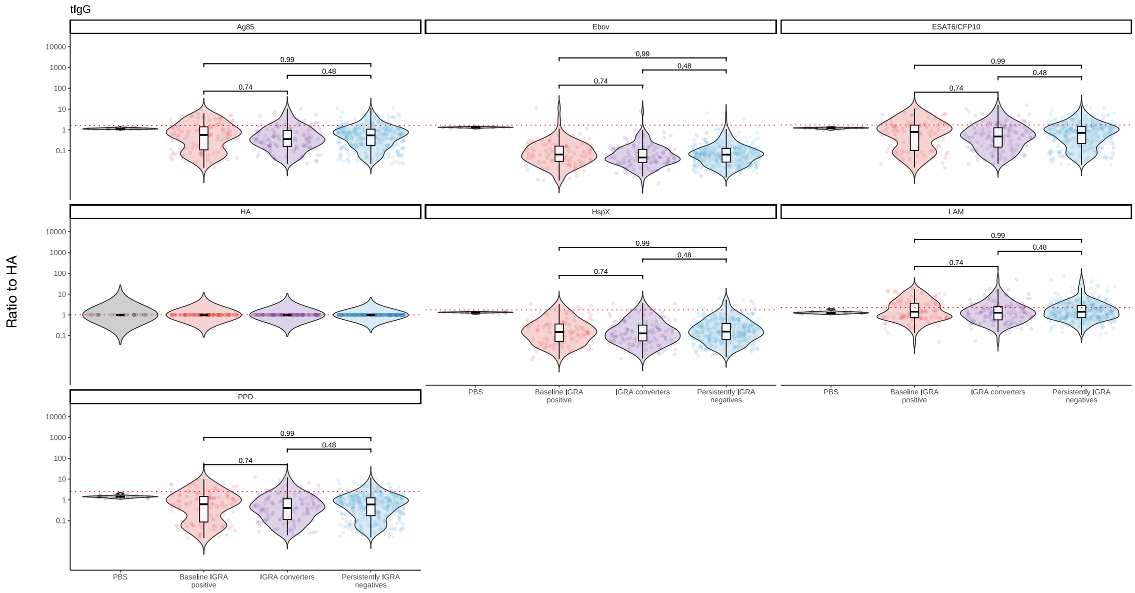
tIgG
Ag85
Ebov
ESAT6/CFP10
HA
HspX
LAM
PPD
Ratio to HA
10000
1000
100
10
1
0.1
0.99
0.48
0.74
PBS
Baseline IGRA positive
IGRA converters
Persistently IGRA negatives

**C**

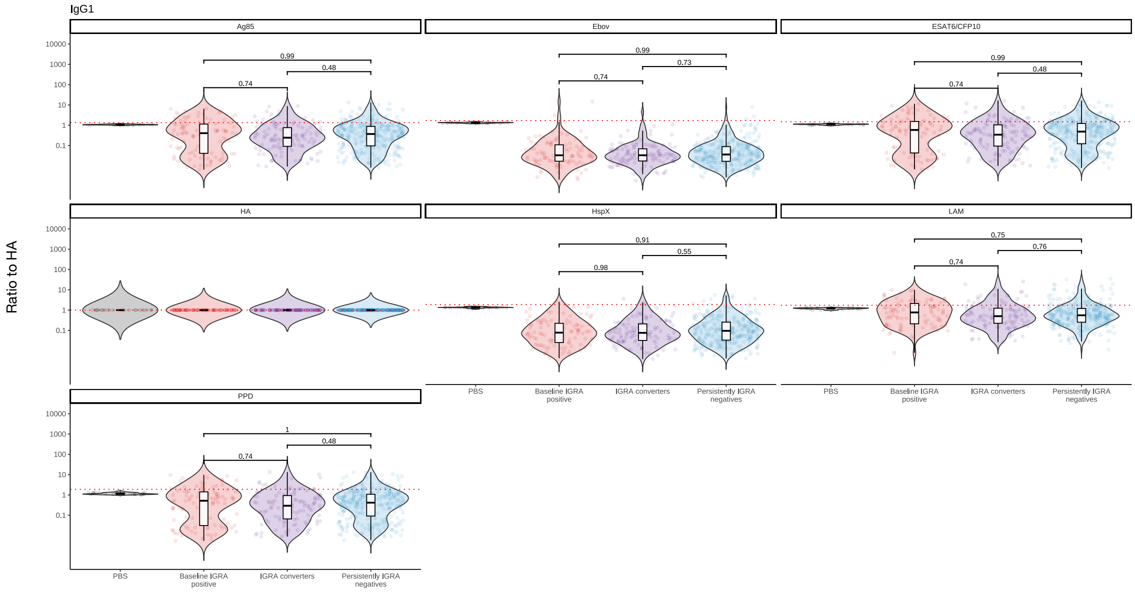
IgG1
Ag85
Ebov
ESAT6/CFP10
HA
HspX
LAM
PPD
Ratio to HA
10000
1000
100
10
1
0.1
0.99
0.48
0.74
0.73
0.91
0.55
0.98
0.75
0.76
1
PBS
Baseline IGRA positive
IGRA converters
Persistently IGRA negatives

**D**

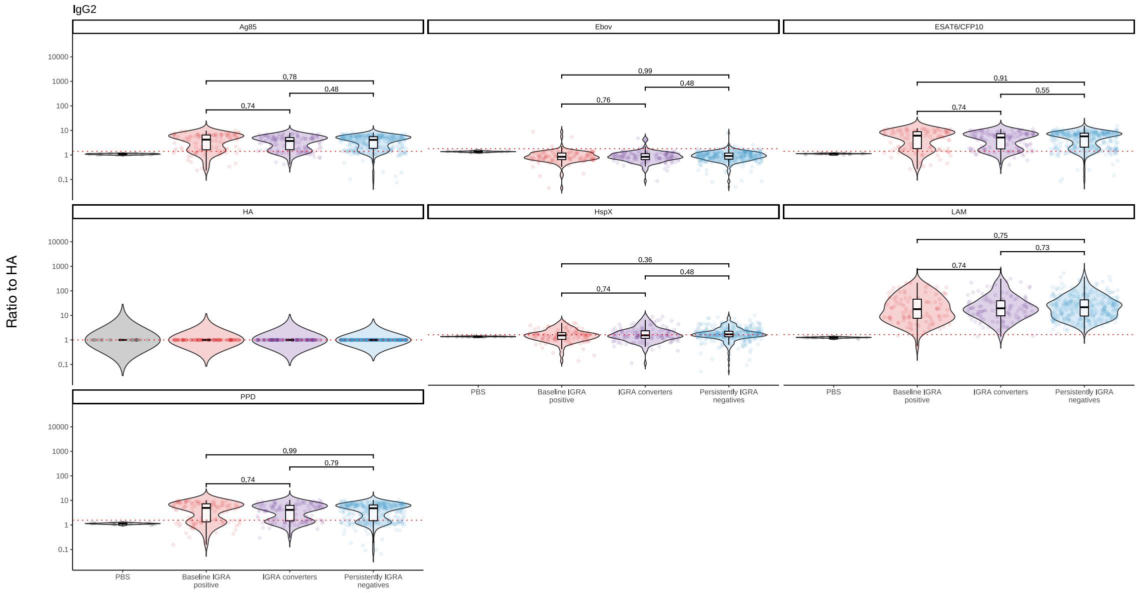
IgG2
Ag85
Ebov
ESAT6/CFP10
HA
HspX
LAM
PPD
Ratio to HA
10000
1000
100
10
1
0.1
0.78
0.48
0.74
0.99
0.48
0.76
0.91
0.55
0.74
0.36
0.48
0.74
0.75
0.73
0.74
0.99
0.79
0.74
PBS
Baseline IGRA positive
IGRA converters
Persistently IGRA negatives

**E**

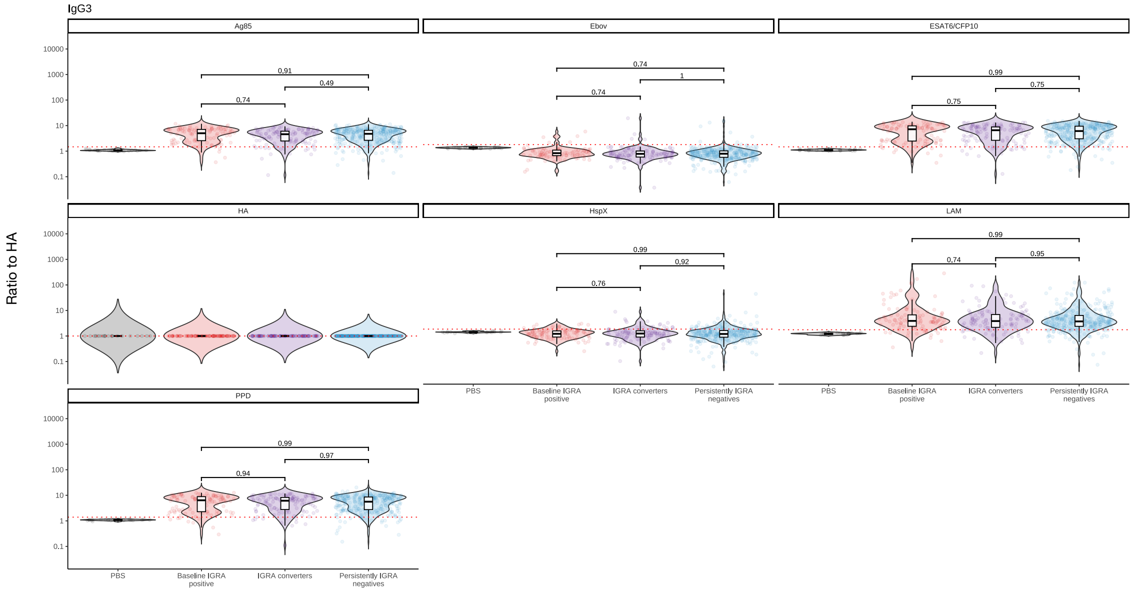
IgG3
Ag85
Ebov
ESAT6/CFP10
HA
HspX
LAM
PPD
Ratio to HA
10000
1000
100
10
1
0.1
0.91
0.49
0.74
0.74
1
0.74
0.99
0.75
0.75
0.99
0.92
0.76
0.99
0.95
0.74
0.99
0.97
0.94
PBS
Baseline IGRA positive
IGRA converters
Persistently IGRA negatives

**F**

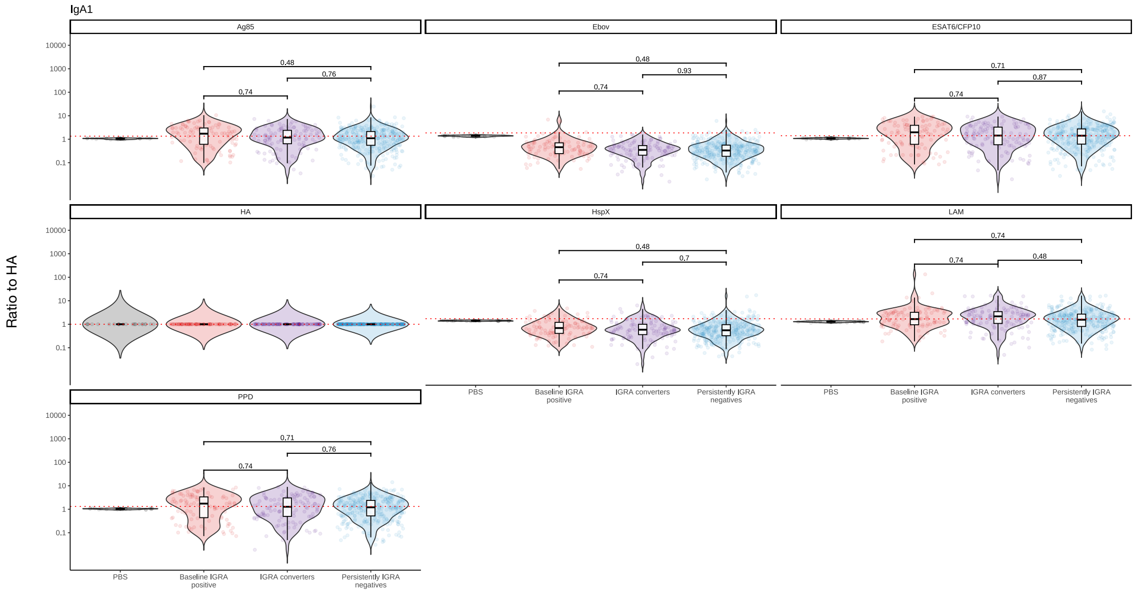
IgA1
Ratio to HA
Ag85
Ebov
ESAT6/CFP10
HA
HspX
LAM
PPD
10000
1000
100
10
1
0,1
0,48
0,76
0,74
0,48
0,93
0,74
0,71
0,87
0,74
0,48
0,7
0,74
0,74
0,74
0,48
0,71
0,76
0,74
PBS
Baseline IGRA positive
IGRA converters
Persistently IGRA negatives

**G**

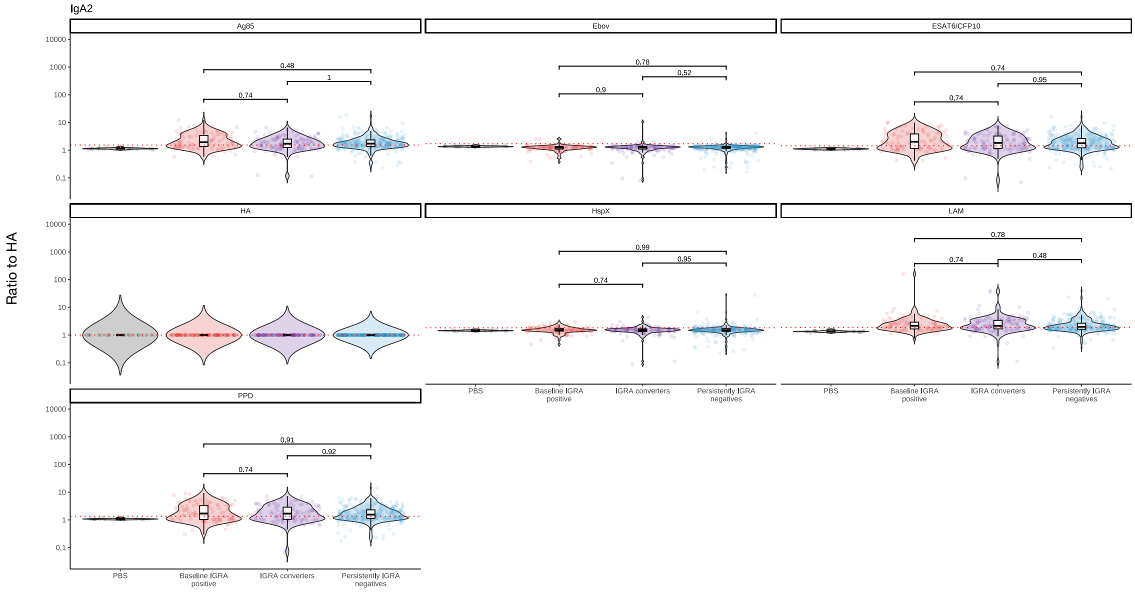
IgA2
Ratio to HA
Ag85
Ebov
ESAT6/CFP10
HA
HspX
LAM
PPD
10000
1000
100
10
1
0,1
0,48
1
0,74
0,78
0,52
0,9
0,74
0,95
0,74
0,99
0,95
0,74
0,78
0,74
0,48
0,91
0,92
0,74
PBS
Baseline IGRA positive
IGRA converters
Persistently IGRA negatives

**H**

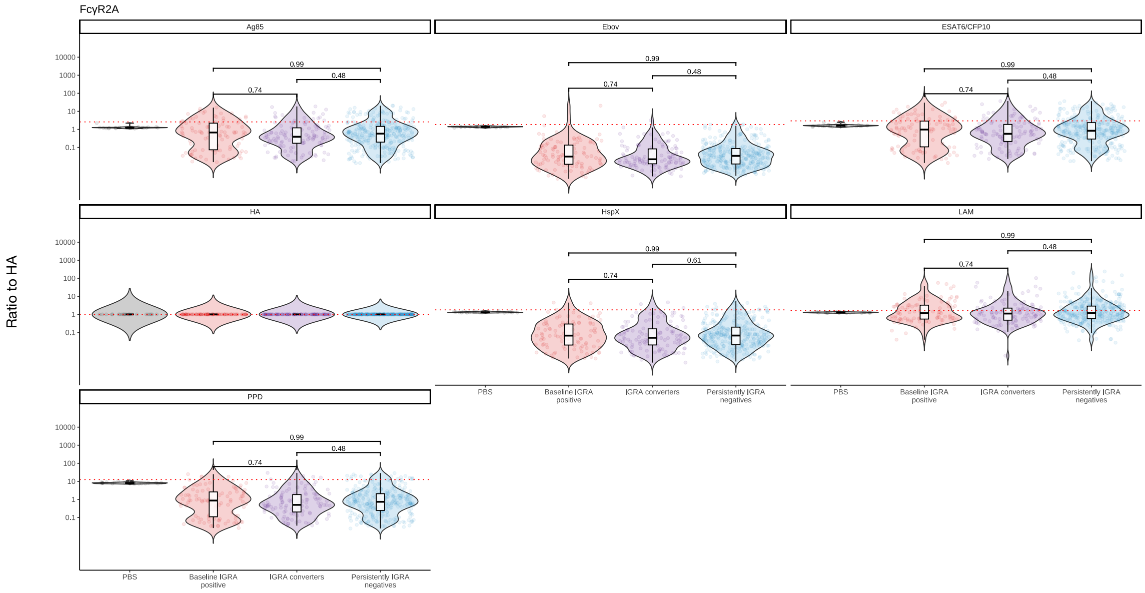
FcγR2A
Ag85
Ebov
ESAT6/CFP10
HA
HspX
LAM
PPD
Ratio to HA
10000
1000
100
10
1
0.1
0.99
0.74
0.48
0.61
PBS
Baseline IGRA positive
IGRA converters
Persistently IGRA negatives

**I**

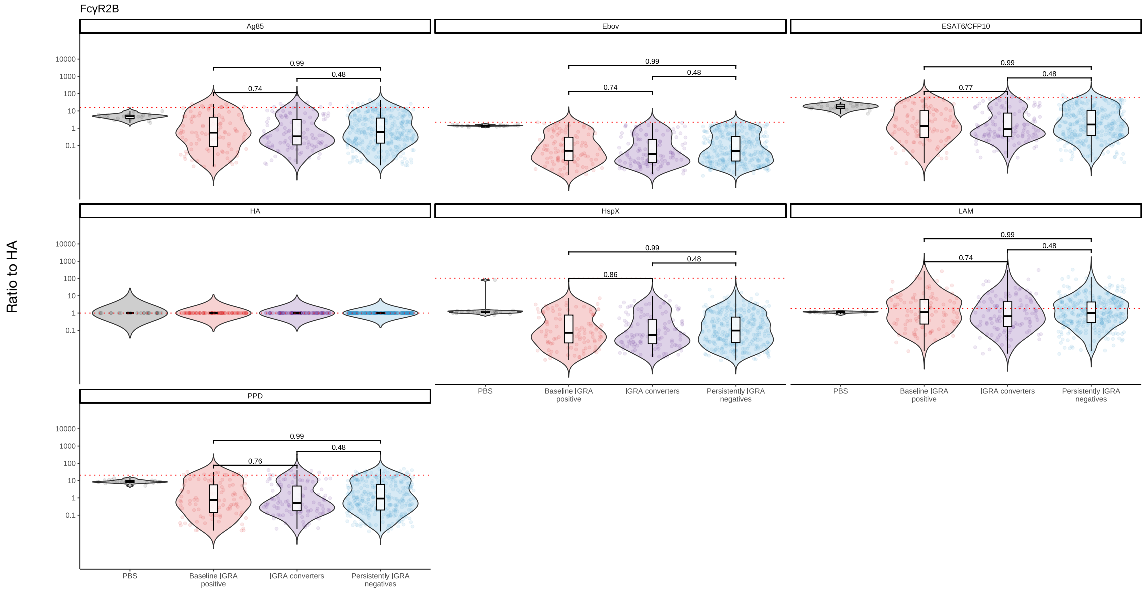
FcγR2B
Ag85
Ebov
ESAT6/CFP10
HA
HspX
LAM
PPD
Ratio to HA
10000
1000
100
10
1
0.1
0.99
0.74
0.48
0.77
0.86
0.76
PBS
Baseline IGRA positive
IGRA converters
Persistently IGRA negatives

**J**

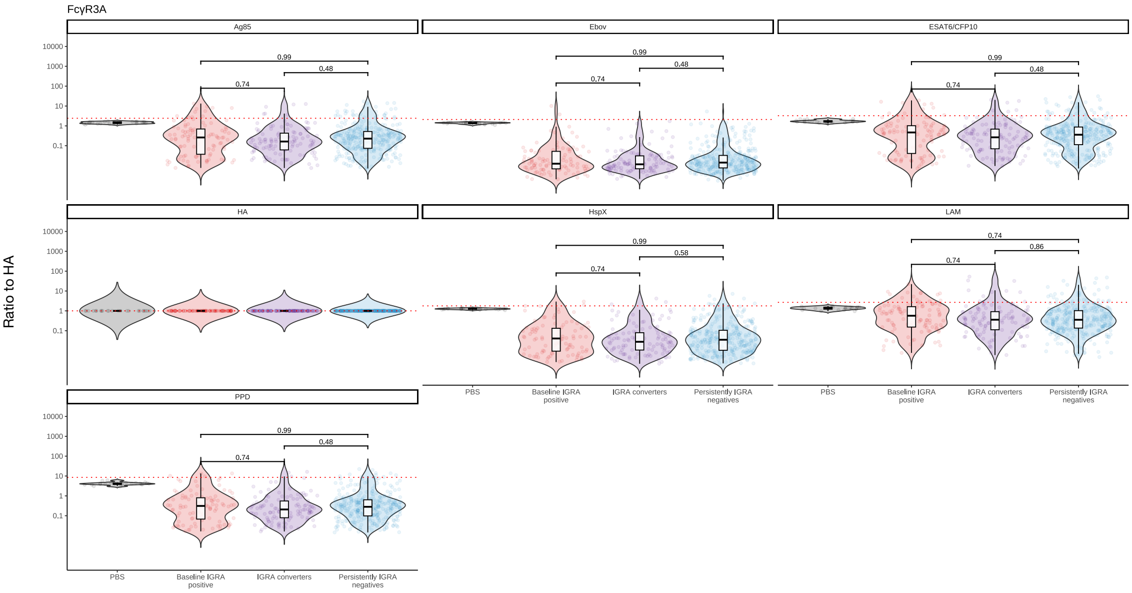
FcγR3A
Ag85
Ebov
ESAT6/CFP10
HA
HspX
LAM
PPD
Ratio to HA
10000
1000
100
10
1
0.1
PBS
Baseline IGRA positive
IGRA converters
Persistently IGRA negatives
0.99
0.48
0.74
0.58
0.86

**K**

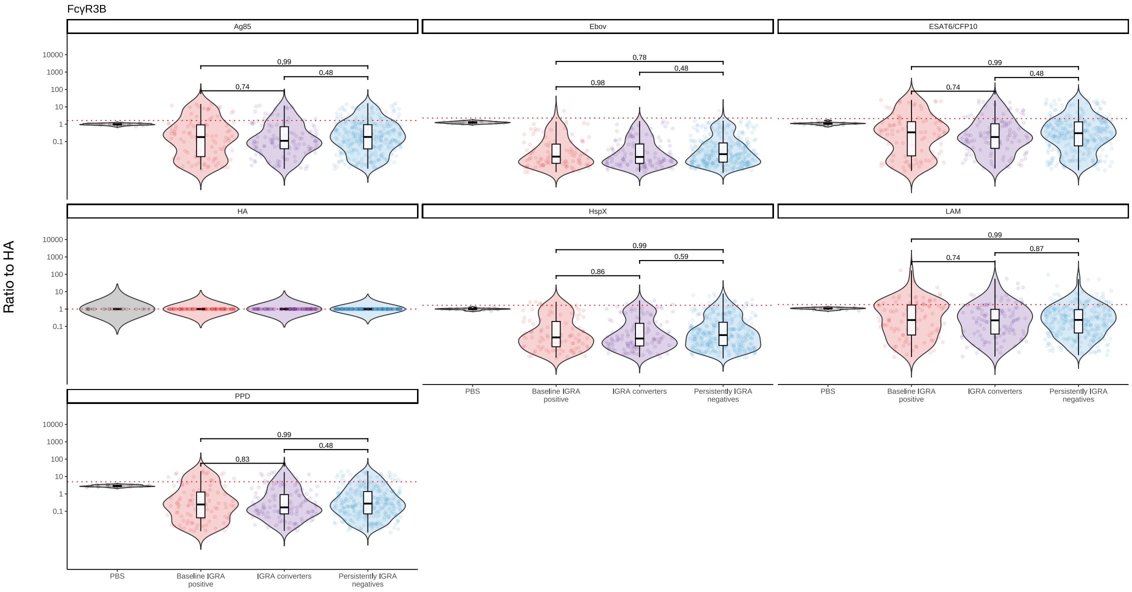
FcγR3B
Ag85
Ebov
ESAT6/CFP10
HA
HspX
LAM
PPD
Ratio to HA
10000
1000
100
10
1
0.1
PBS
Baseline IGRA positive
IGRA converters
Persistently IGRA negatives
0.99
0.48
0.74
0.78
0.98
0.86
0.59
0.87
0.83

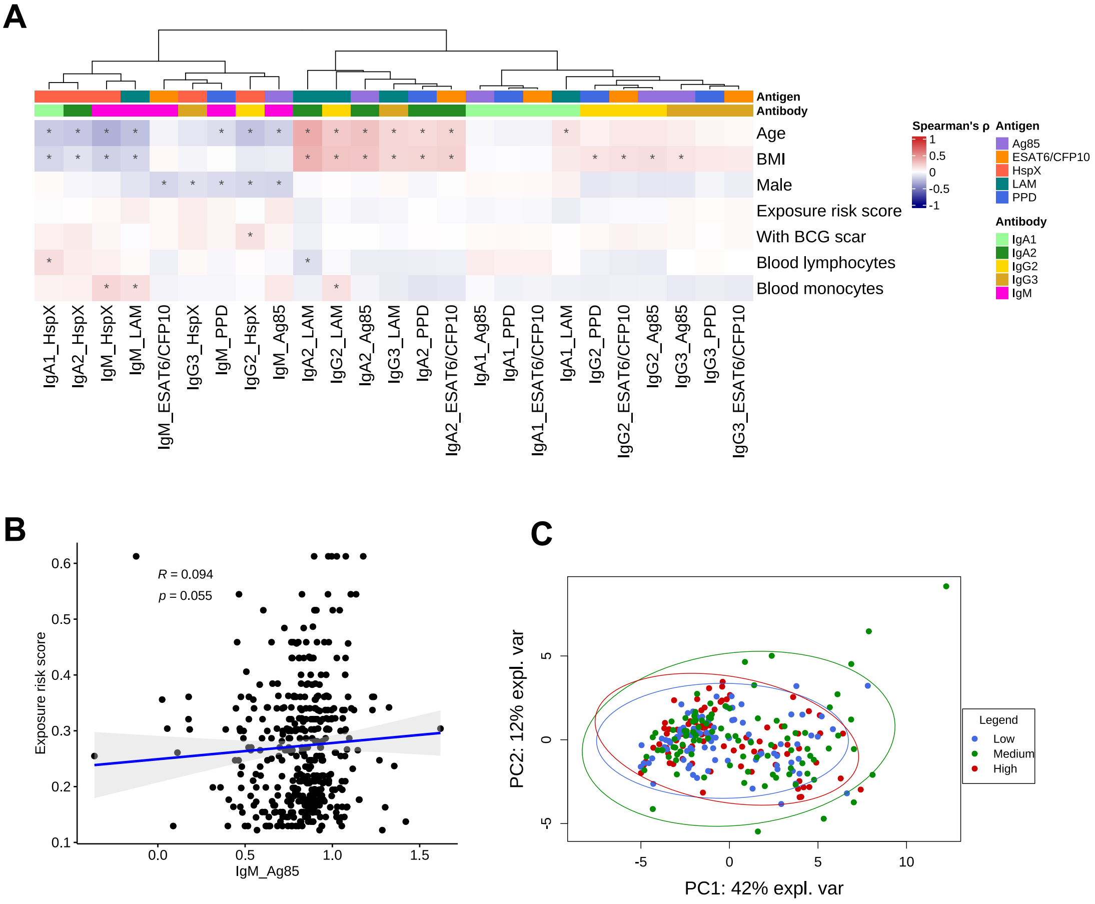


**Supplementary Figure 5. Correlation of antibody profiles with tuberculosis exposure and other subject characteristics in IGRA-negative individuals.**

Heatmap of the correlation between baseline antibody levels and subject characteristics, based on Spearman's rho (FDR <0.1, *) in IGRA-negative individuals (**A**). Correlation plot of Ag85-specific IgM and exposure risk score (**B**)**.** PCA of antibody measurements stratified by the level of exposure (Low = 1$^{st}$ tertile, Medium = 2$^{nd}$ tertile, High = 3$^{rd}$ tertile) (**C**).

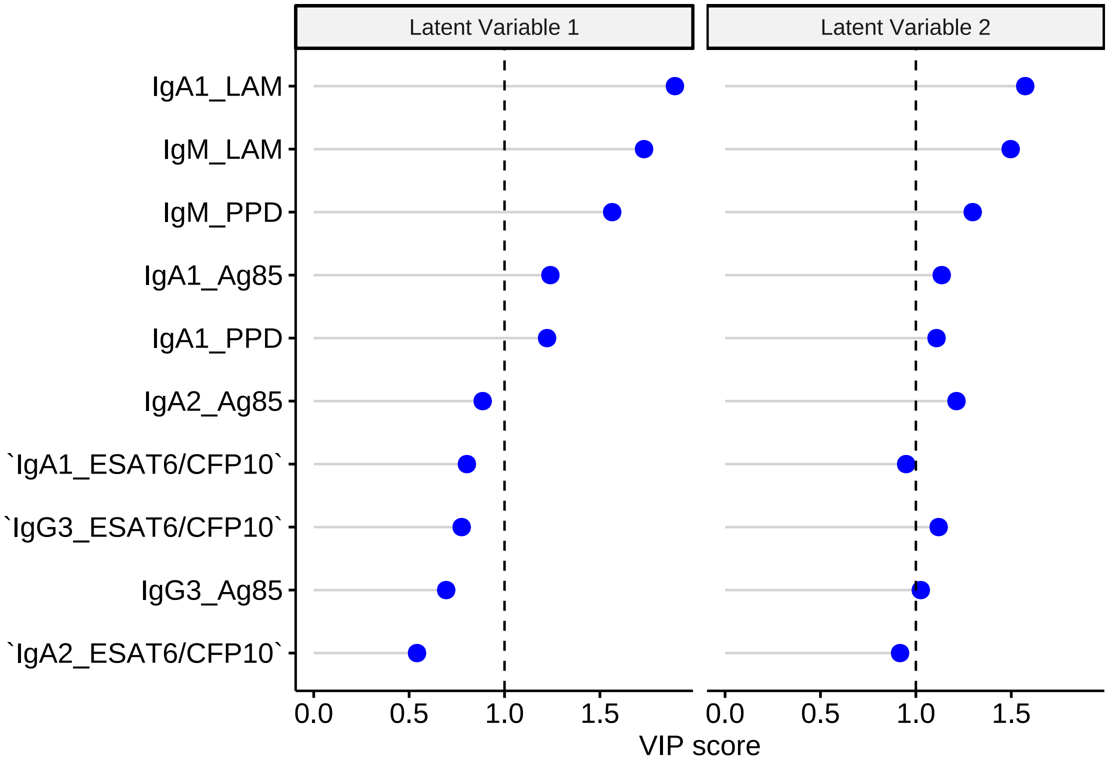


**Supplementary Figure 6. Variable importance in projection score of PLS-DA in IGRA-negative individuals.**

Variable importance in projection (VIP) coefficients of the PLS-DA in IGRA-negative individuals. The VIP score represent the relative importance of each antibody to explain IGRA converters and persistently IGRA-negatives group. The most relevant features to explain the difference between groups have VIP score > 1.

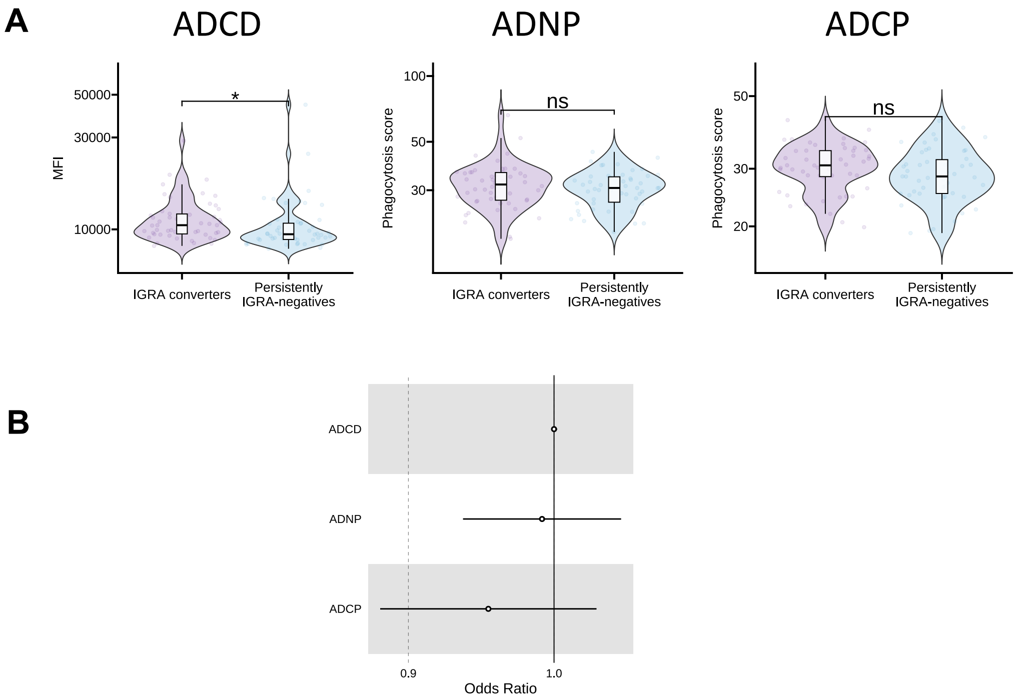


**Supplemental Figure 7. Antibody functionality in IGRA-converters and persistently IGRA-negative individuals.**

Using strict IGRA cutoff criteria (<0.15 IU/mL and >0.70 IU/mL), antibody dependent complement deposition, antibody-dependent cellular phagocytosis, and antibody-dependent neutrophil phagocytosis were compared between persistently IGRA-negatives (N = 50) and IGRA converters (N = 50). In Mann-Whitney U test, IGRA converters showed more antibody dependent complement deposition compared to persistently IGRA-negatives (FDR=0.031) (**A**), but in a logistic regression model adjusting for age, sex, BMI and correcting for multiple testing, there was no significant association between antibody function and IGRA conversion (open circle, FDR > 0.1) (**B**).

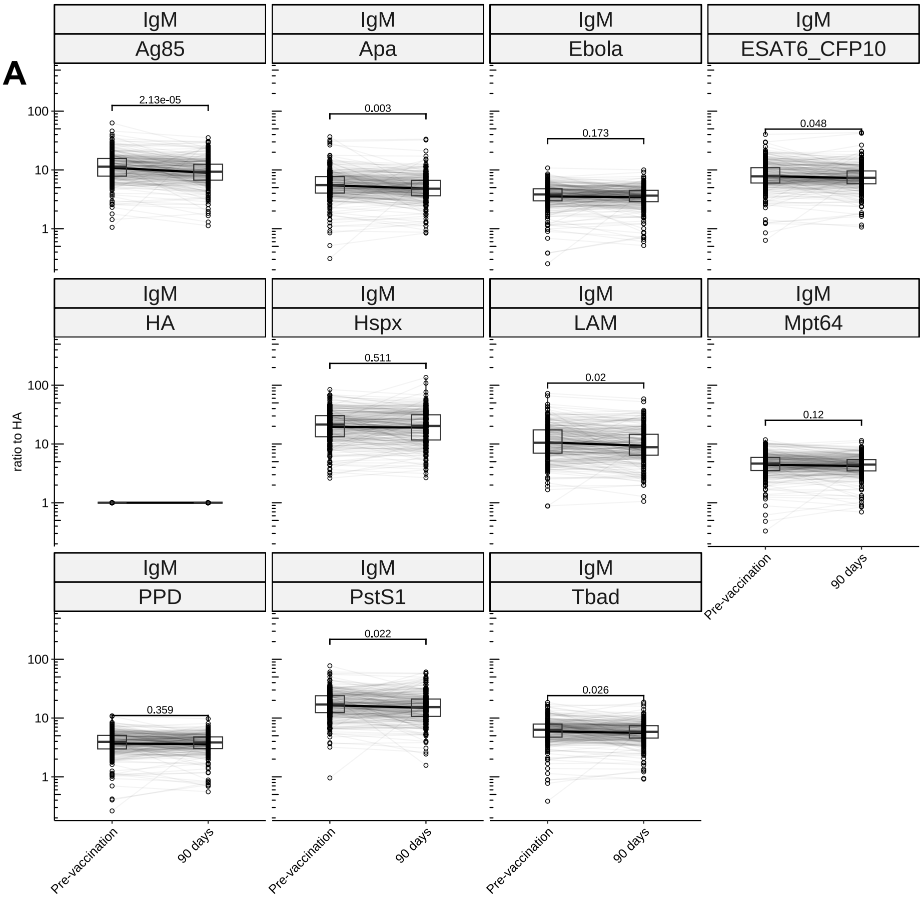


**Supplemental Figure 8**. **Effect of BCG vaccination on *Mtb* antigen-specific antibodies**

Concentrations of IgM (**A**), total IgG, IgG1 (**B**), IgG2, IgG3 (**C**), FcγR2A, FcγR3A (**D**) at baseline and 90 days after vaccination. (FDR<0.1, <0.05, <0.01, <0.001; *, **, ***, ****)

B

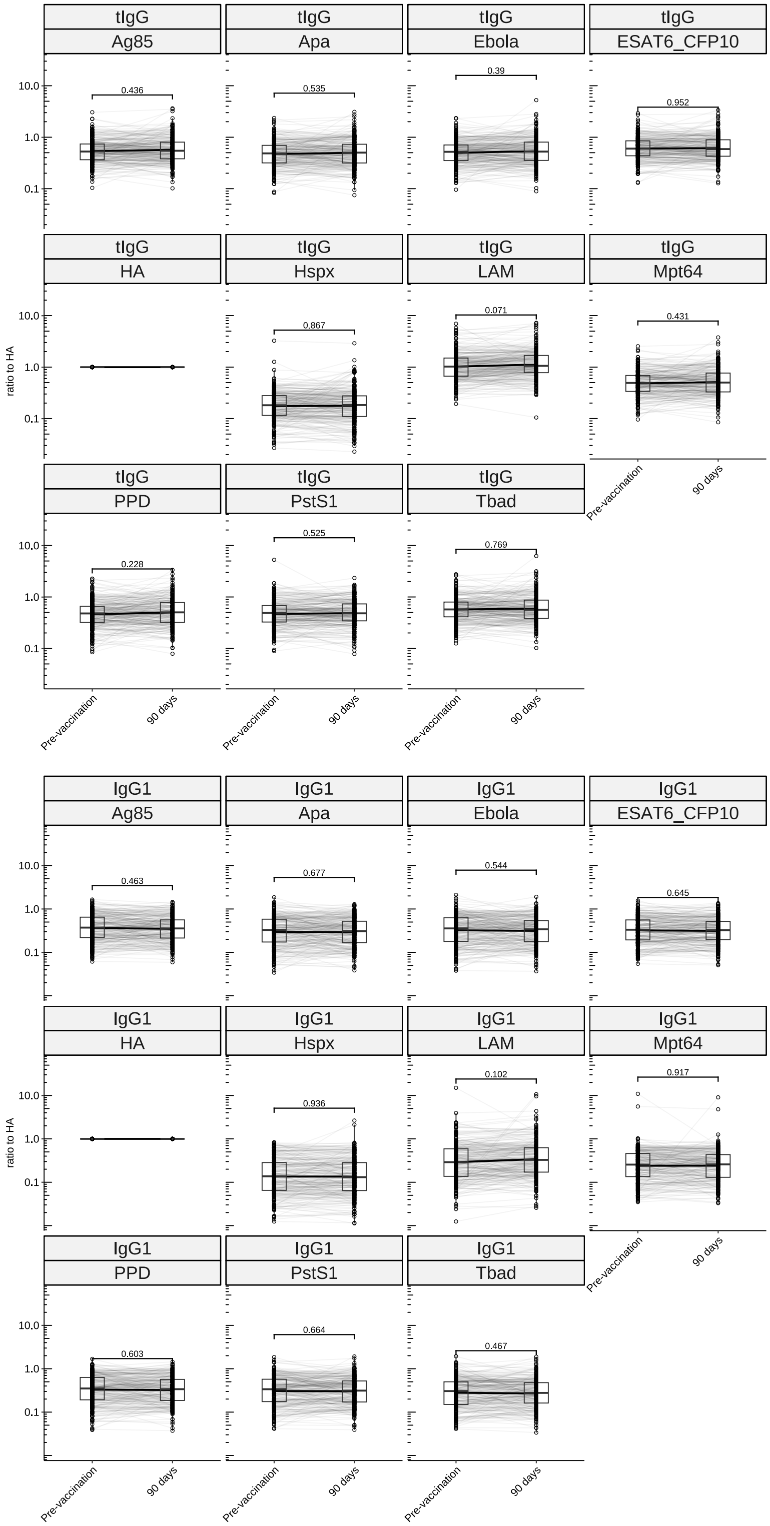
tIgG
Ag85
Apa
Ebola
ESAT6_CFP10
HA
Hspx
LAM
Mpt64
PPD
PstS1
Tbad
ratio to HA
Pre-vaccination
90 days
0.436
0.535
0.39
0.952
0.867
0.071
0.431
0.228
0.525
0.769
IgG1
0.463
0.677
0.544
0.645
0.936
0.102
0.917
0.603
0.664
0.467

C

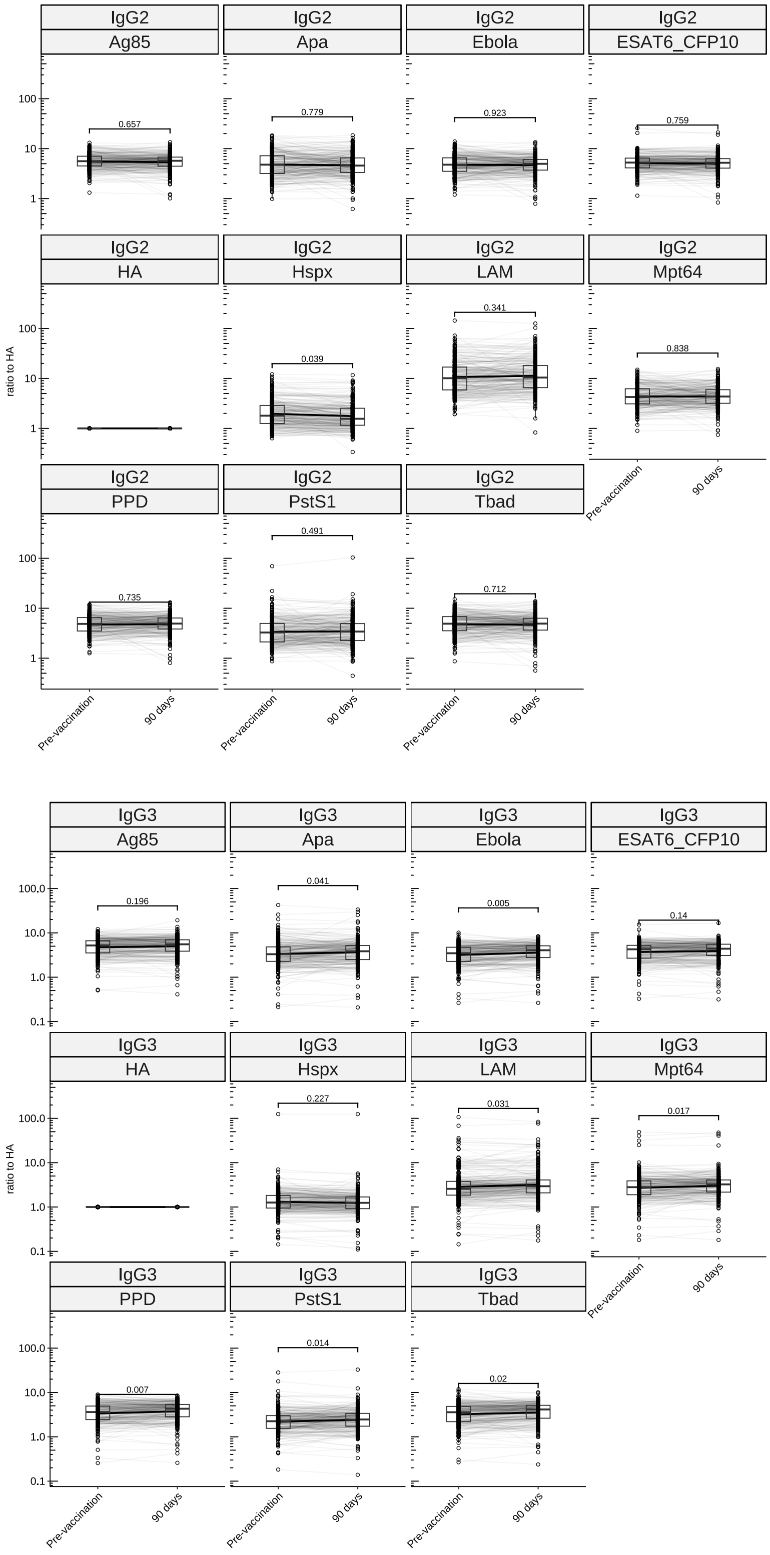

IgG2
Ag85
Apa
Ebola
ESAT6_CFP10
HA
Hspx
LAM
Mpt64
PPD
PstS1
Tbad
ratio to HA
0.657
0.779
0.923
0.759
0.039
0.341
0.838
0.735
0.491
0.712
Pre-vaccination
90 days
IgG3
Ag85
Apa
Ebola
ESAT6_CFP10
HA
Hspx
LAM
Mpt64
PPD
PstS1
Tbad
ratio to HA
0.196
0.041
0.005
0.14
0.227
0.031
0.017
0.007
0.014
0.02
Pre-vaccination
90 days

**D**

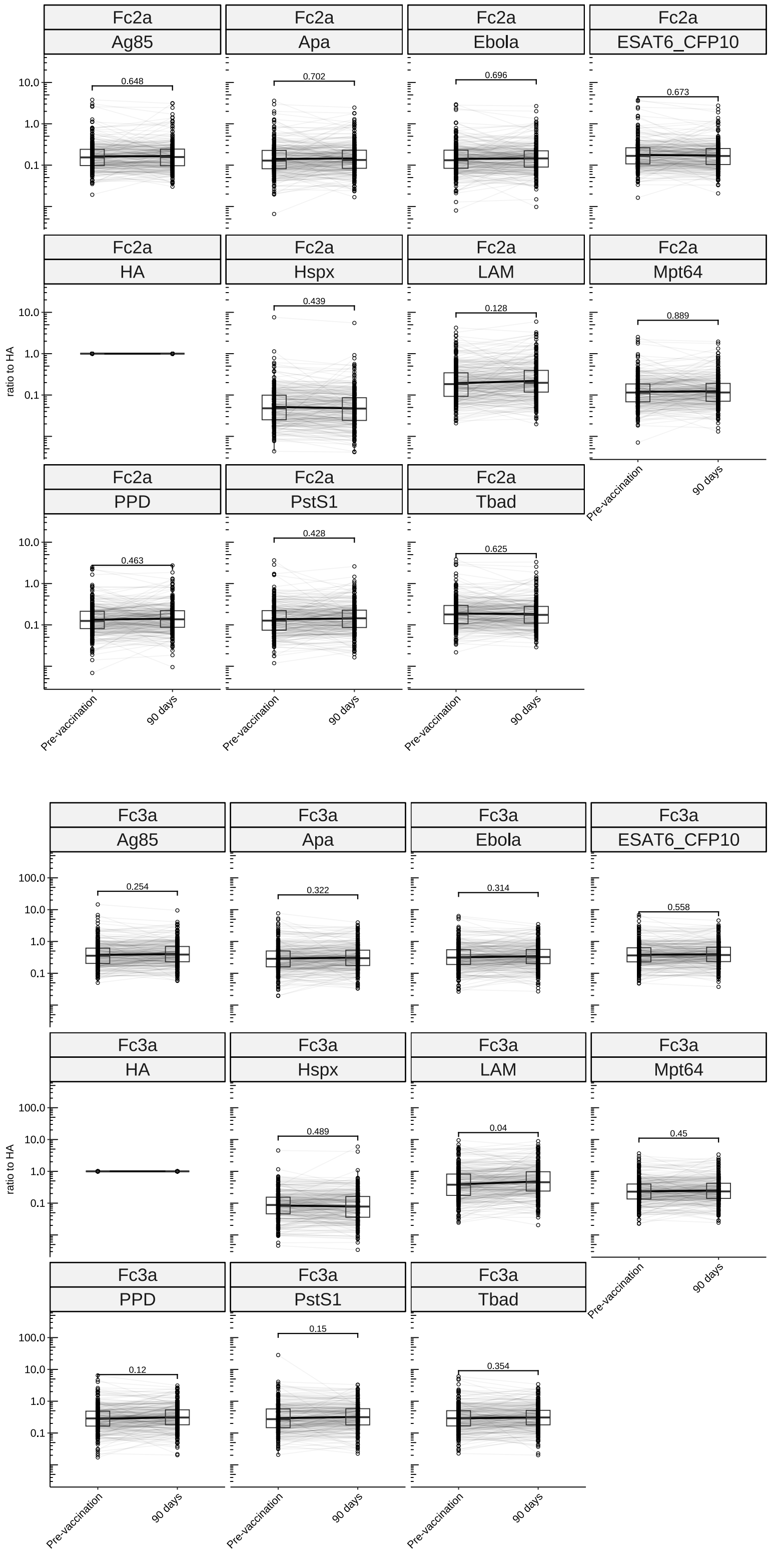

Fc2a Ag85
0.648
Fc2a Apa
0.702
Fc2a Ebola
0.696
Fc2a ESAT6_CFP10
0.673
Fc2a HA
Fc2a Hspx
0.439
Fc2a LAM
0.128
Fc2a Mpt64
0.889
Fc2a PPD
0.463
Fc2a PstS1
0.428
Fc2a Tbad
0.625
ratio to HA
10.0
1.0
0.1
Pre-vaccination
90 days
Fc3a Ag85
0.254
Fc3a Apa
0.322
Fc3a Ebola
0.314
Fc3a ESAT6_CFP10
0.558
Fc3a HA
Fc3a Hspx
0.489
Fc3a LAM
0.04
Fc3a Mpt64
0.45
Fc3a PPD
0.12
Fc3a PstS1
0.15
Fc3a Tbad
0.354
100.0
