# Supplementary material for: Immune correlates of early clearance of Mycobacterium tuberculosis among tuberculosis household contacts in Indonesia
Source: Nat Commun. 2025 Jan 2;16:309. doi: 10.1038/s41467-024-55501-6 (PMC11695729; doi:10.1038/s41467-024-55501-6)
Supplement: Supplementary file 3 — Reporting Summary [file 41467_2024_55501_MOESM3_ESM.pdf]

nature portfolio

Corresponding author(s): Prof. dr. Reinout van Crevel

Last updated by author(s): Nov 22, 2024

Reporting Summary

Nature Portfolio wishes to improve the reproducibility of the work that we publish. This form provides structure for consistency and transparency in reporting. For further information on Nature Portfolio policies, see our Editorial Policies and the Editorial Policy Checklist.

Statistics

For all statistical analyses, confirm that the following items are present in the figure legend, table legend, main text, or Methods section.

n/a	Confirmed	
☐	☒	The exact sample size (*n*) for each experimental group/condition, given as a discrete number and unit of measurement
☐	☒	A statement on whether measurements were taken from distinct samples or whether the same sample was measured repeatedly
☐	☒	The statistical test(s) used AND whether they are one- or two-sided<br>*Only common tests should be described solely by name; describe more complex techniques in the Methods section.*
☐	☒	A description of all covariates tested
☐	☒	A description of any assumptions or corrections, such as tests of normality and adjustment for multiple comparisons
☐	☒	A full description of the statistical parameters including central tendency (e.g. means) or other basic estimates (e.g. regression coefficient) AND variation (e.g. standard deviation) or associated estimates of uncertainty (e.g. confidence intervals)
☐	☒	For null hypothesis testing, the test statistic (e.g. *F*, *t*, *r*) with confidence intervals, effect sizes, degrees of freedom and *P* value noted<br>*Give P values as exact values whenever suitable.*
☐	☒	For Bayesian analysis, information on the choice of priors and Markov chain Monte Carlo settings
☐	☒	For hierarchical and complex designs, identification of the appropriate level for tests and full reporting of outcomes
☒	☐	Estimates of effect sizes (e.g. Cohen's *d*, Pearson's *r*), indicating how they were calculated

Our web collection on statistics for biologists contains articles on many of the points above.

Software and code

Policy information about availability of computer code

Data collection	Flow cytometry data were collected using Cell Quest Pro software (BD Bioscience)
Data analysis	All data analysis were done in R 4.3.2 within Rstudio environment. The initial flow cytometry data were analysed using using FlowJo version 10.4 (TreeStar), while the analysis using extracted cell number data for this study were done using R and Rstudio.

For manuscripts utilizing custom algorithms or software that are central to the research but not yet described in published literature, software must be made available to editors and reviewers. We strongly encourage code deposition in a community repository (e.g. GitHub). See the Nature Portfolio guidelines for submitting code & software for further information.

Data

Policy information about availability of data

All manuscripts must include a data availability statement. This statement should provide the following information, where applicable:

- Accession codes, unique identifiers, or web links for publicly available datasets
- A description of any restrictions on data availability
- For clinical datasets or third party data, please ensure that the statement adheres to our policy

The data supporting the findings of this study are available in the accompanying Supplementary Information

Research involving human participants, their data, or biological material

Policy information about studies with human participants or human data. See also policy information about sex, gender (identity/presentation), and sexual orientation and race, ethnicity and racism.

Reporting on sex and gender

only the terms sex (biological attributes) were used in this study

Reporting on race, ethnicity, or other socially relevant groupings

Not applicable. The only race/ethnicity we stated here was that INFECT was done in Indonesia and 300BCG was done in the Netherlands. The difference that we highlighted in our manuscript is that the exposure to M. tuberculosis is different in these two locations

Population characteristics

INFECT Baseline IGRA positive vs IGRA negative [Median (IQR); n (%)]

contact characteristics	Baseline IGRA-positive (N = 780)	Baseline IGRA-negative (N = 433)	Pvalue
Age	31 (17 –47)	22 (12 –39)	<0.001
Female sex	58%	53%	0.089
Presence of BCG scar	78%	84%	0.013
Current and previous smoking	35%	31%	0.27
BMI, kg/m2	21.6 (18.0 –25.4)	20.2 (16.8 –24.4)	0.001
Diabetes	3.20%	3.90%	0.41

INFECT IGRA converters vs persistently IGRA negatives [Median (IQR); n (%)]

	IGRA converters (N = 116)	Persistently IGRA-negatives (N = 317)	Pvalue
Age	23 (15–36)	22 (12–40)	0.8
Female sex	51%	54%	0.6
Presence of BCG scar	74%	87%	<0.001
Current and previous smoking	36%	29%	0.2
BMI, kg/m2	21.0 (17.7–24.8)	20.0 (16.7–24.3)	0.12
Diabetes	5.2%	3.5%	0.5

300BCG BCG vaccinated Dutch adult

	N=298	
Age	23 (18 –71)	[Median (range)]
Female sex	56%	
BMI, kg/m2	22.15 (20.80 –23.62)	[Median (IQR)]

these characteristics are included in an Excel sheet provided as supplementary information

Recruitment

INFECT: Household contacts of newly diagnosed smear-positive TB patients that had received <2 weeks of TB treatment were eligible if they were older than 5 years and had had no previous TB.

300BCG: The BCG vaccination cohort (300BCG) recruited volunteers of Western European ancestry between April 2017 and June 2018 at the Radboud University Medical Center.

Ethics oversight

INFECT: Health Research Ethics Committee of Universitas Padjadjaran Indonesia (14/UN6.C2.1.2/KEPK/PN/2014) and the Southern Health and Disability Ethics Committee New Zealand (13/STH/132).

300BCG: Arnhem-Nijmegen Medical Ethical Committee (NL58553.091.16)

Note that full information on the approval of the study protocol must also be provided in the manuscript.

Field-specific reporting

Please select the one below that is the best fit for your research. If you are not sure, read the appropriate sections before making your selection.

☒ Life sciences ☐ Behavioural & social sciences ☐ Ecological, evolutionary & environmental sciences

For a reference copy of the document with all sections, see nature.com/documents/nr-reporting-summary-flat.pdf

Life sciences study design

All studies must disclose on these points even when the disclosure is negative.

Sample size

Sample size were determined by the availability of the sample/data.
INFECT Flow cytometry: Among baseline IGRA-negative, a subset of 102 participants agreed to have their blood collected at week 2 and week 14 after enrollment.

INFECT Innate ex vivo cytokine: all participants have their blood collected for whole blood ex-vivo cytokine stimulation.

	INFECT Olink inflammatory: A subset of participants were selected using strict IGRA-cut offs classification with roughly the same proportion in IGRA converters and persistently IGRA negative group with total INFECT sample with baseline IGRA-negative (around 1:2.7).<br><br>300BCG cytokine and antibody: all participants were included
Data exclusions	INFECT flow cytometry analysis: exclusion was done based on the availability of measurements. Sample without measurements at both week 2 and week 14 were excluded<br><br>INFECT innate cytokine analysis: Contaminated samples (presence of IL-6 without any stimulation; mostly because of a contaminated batch of blood collection tubes) were excluded<br><br>INFECT Olink inflammatory proteins: Samples with high hemolysis (>7.5g/L) were excluded<br><br>300BCG cytokines and antibody: Participants were excluded if they had been using systemic medications (excluding oral contraceptives or acetaminophen), antibiotics within three months prior to the study, a previous BCG vaccination, a history of tuberculosis, any feverish illness in the four weeks preceding the study, any vaccinations in the three months before the study, or had a medical history indicating immunodeficiency. For the analysis in the study, only measurements from prevaccination and 90 days post-vaccination were included. Measurements from 14 days post-vaccination was excluded
Replication	Flow cytometry, ex-vivo cytokines, and Olink inflammatory proteins data measurements were only done once due to sample limitation. Luminex data was gathered twice in technical duplicate.
Randomization	In INFECT ex vivo cytokines, no randomization were done. Age, sex, and BMI as covariates were controlled in the regression model.<br><br>In INFECT Olink inflammatory proteins, samples were sex and age matched and have roughly the same proportion of sample in IGRA converters and persistently IGRA negative (1:2.6) with total INFECT sample with baseline IGRA-negative (1:2.7). In addition, age, sex, and BMI as covariates were controlled in the regression model.<br><br>In INFECT antibody, for the selection of N=100 baseline IGRA positive, samples were selected by age and sex matching. for the baseline IGRA negative, all samples were collected. Age, sex, and BMI as covariates were controlled in the regression model in the analysis.<br><br>Randomization were not done in 300BCG
Blinding	Ex vivo cytokines, Olink inflammatory proteins, luminex and Fc effector functions data was gathered blinded. Analysis was performed after collection of aforementioned data and unblinding.

Reporting for specific materials, systems and methods

We require information from authors about some types of materials, experimental systems and methods used in many studies. Here, indicate whether each material, system or method listed is relevant to your study. If you are not sure if a list item applies to your research, read the appropriate section before selecting a response.

Materials & experimental systems

n/a	Involved in the study	
☐	☒	Antibodies
☒	☐	Eukaryotic cell lines
☒	☐	Palaeontology and archaeology
☒	☐	Animals and other organisms
☒	☐	Clinical data
☒	☐	Dual use research of concern
☒	☐	Plants

Methods

n/a	Involved in the study	
☒	☐	ChIP-seq
☐	☒	Flow cytometry
☒	☐	MRI-based neuroimaging

Antibodies

Antibodies used	CD14 AlexaFluor 488<br>CD16 PE<br>HLADR PerCP<br>CXCR4 APC<br>CD3 AlexaFluor 488<br>Vα7.2 PE<br>CD56 PerCP<br>CD161 APC<br>Vα24-Jα18 PE<br>Vδ2 PerCP<br>γδ TCR APC

	(all from Biolegend).
Validation	Each antibody used had a validated technical data sheet as per manufacturer's website showing positive staining, and titrated in our laboratory prior to their use.

Plants

Seed stocks	*Report on the source of all seed stocks or other plant material used. If applicable, state the seed stock centre and catalogue number. If plant specimens were collected from the field, describe the collection location, date and sampling procedures.*
Novel plant genotypes	*Describe the methods by which all novel plant genotypes were produced. This includes those generated by transgenic approaches, gene editing, chemical/radiation-based mutagenesis and hybridization. For transgenic lines, describe the transformation method, the number of independent lines analyzed and the generation upon which experiments were performed. For gene-edited lines, describe the editor used, the endogenous sequence targeted for editing, the targeting guide RNA sequence (if applicable) and how the editor was applied.*
Authentication	*Describe any authentication procedures for each seed stock used or novel genotype generated. Describe any experiments used to assess the effect of a mutation and, where applicable, how potential secondary effects (e.g. second site T-DNA insertions, mosiacism, off-target gene editing) were examined.*

Flow Cytometry

Plots

Confirm that:

- [ ] The axis labels state the marker and fluorochrome used (e.g. CD4-FITC).
- [ ] The axis scales are clearly visible. Include numbers along axes only for bottom left plot of group (a 'group' is an analysis of identical markers).
- [ ] All plots are contour plots with outliers or pseudocolor plots.
- [x] A numerical value for number of cells or percentage (with statistics) is provided.

Methodology

Sample preparation	For immunophenotyping by flow cytometry, 200 µL of heparinized blood was mixed with 30 µL of 123Count eBeads (eBioscience) for each antibody panel to allow an absolute cell count. Samples were then washed once in phosphate-buffered saline (PBS) and stained with one of 3 antibody panels in icecold fluorescence activated cell sorting (FACS) buffer (PBS, 0.5% bovine serum albumin, 0.1% sodium azide).<br>Panel 1: CD14 AlexaFluor 488, CD16 PE, HLADR PerCP, CXCR4 APC;<br>panel 2: CD3 AlexaFluor 488, Vα7.2 PE, CD56 PerCP, CD161 APC;<br>panel 3: CD3 AlexaFluor 488, Vα24-Jα18 PE, Vδ2 PerCP, γδ TCR APC (all from Biolegend).<br><br>Blood was stained at 4°C for 20 minutes. Samples were then fixed and lysed using 1× FACS lysing solution (BD Biosciences), with incubation at room temperature for 12 minutes, followed by 2 washes in FACS buffer.
Instrument	FACSCalibur (BD Bioscience)
Software	Data were collected using Cell Quest Pro software (BD Bioscience) and data were analyzed using FlowJo version 10.4 (TreeStar).
Cell population abundance	Use of 123Count eBeads (eBioscience) for each antibody panel to allow absolute cell counting
Gating strategy	Gating strategies and flow cytometry plots are located in Supplementary Information of Verral et al., 2020 (PMID: 30958547)

- [ ] Tick this box to confirm that a figure exemplifying the gating strategy is provided in the Supplementary Information.
